# The impact of cannabinoids on inflammasome signaling in HIV-1 infection

**DOI:** 10.1515/nipt-2023-0002

**Published:** 2023-03-25

**Authors:** Alice K. Min, Aislinn M. Keane, Matthew Paltiel Weinstein, Talia H. Swartz

**Affiliations:** Division of Infectious Diseases, Department of Medicine, Icahn School of Medicine at Mount Sinai, New York, NY, USA

**Keywords:** cannabinoids, HIV-1, immune activation, infection, inflammasome

## Abstract

Human immunodeficiency virus type 1 (HIV-1) is a chronic disease that afflicts over 38 million people worldwide without a known cure. The advent of effective antiretroviral therapies (ART) has significantly decreased the morbidity and mortality associated with HIV-1 infection in people living with HIV-1 (PWH), thanks to durable virologic suppression. Despite this, people with HIV-1 experience chronic inflammation associated with co-morbidities. While no single known mechanism accounts for chronic inflammation, there is significant evidence to support the role of the NLRP3 inflammasome as a key driver. Numerous studies have demonstrated therapeutic impact of cannabinoids, including exerting modulatory effects on the NLRP3 inflammasome. Given the high rates of cannabinoid use in PWH, it is of great interest to understand the intersecting biology of the role of cannabinoids in HIV-1-associated inflammasome signaling. Here we describe the literature of chronic inflammation in people with HIV, the therapeutic impact of cannabinoids in PWH, endocannabinoids in inflammation, and HIV-1-associated inflammation. We describe a key interaction between cannabinoids, the NLRP3 inflammasome, and HIV-1 viral infection, which supports further investigation of the critical role of cannabinoids in HIV-1 infection and inflammasome signaling.

## Chronic inflammation in HIV-1 infection

Human immunodeficiency virus type 1 (HIV-1) is a global public health issue affecting more than 38 million people worldwide [1]. There is no known cure for HIV-1. However, effective antiretroviral therapies (ART) could reduce a person’s viral load to undetectable levels. Durable virologic suppression has significantly reduced the morbidity and mortality in people with HIV-1 (PWH). In contrast, conditions related to aging, including neurocognitive decline, cardiovascular disease, clotting disorders, osteoporosis, and non-AIDS-related cancers, occur at higher rates and lower ages in PWH [2–6]. This accelerated aging process in PWH is thought to be multifactorial. Still, they are mainly attributed to chronic systemic inflammation due to immune activation and immunosenescence stemming from persistent low-level viremia in specific anatomical niches where ART cannot reach [4–8].

Previous studies have demonstrated that PWH on suppressive ART exhibit a pro-inflammatory immune profile even when viral loads are well below the detection level [4, 9], [10], [11], [12], [13], [14], [15], [16], [17]. HIV-1 replication persists below the detection threshold in unique bio-niches [18–21]. Several studies have demonstrated that PWH has elevated pro-inflammatory biomarkers compared to people without HIV-1. In one study, patients with HIV-1 RNA levels below 400 copies/mL exhibited upregulation of high sensitivity C-reactive protein (CRP), D-dimer, and cystatin C levels [22]. Similarly, in another longitudinal analysis, PWH on suppressive ART with HIV-1 RNA level of fewer than 50 copies/mL harbored pro-inflammatory immune profiles [23]. Thus, even in patients on long-term ART therapy with controlled viral loads, inflammatory processes persist at rates conducive to the emergence of inflammation-related comorbidities. Analyses of inflammatory biomarkers in MSM PWH populations yielded similar findings where they exhibited monocyte and macrophage upregulation despite suppressed viremia [24].

During acute HIV-1 infection, activated CD4^+^ T-cells are a significant target of HIV-1 [25–27]. The peripheral lymphoid tissues are seeded and are important sites of HIV-1 replication, including the gut-associated lymphoid tissue (GALT) [20]. The gut, which houses approximately 70% of CD4^+^ T-cells, has been widely acknowledged as a primary site of HIV-1 pathogenesis and persistent viremia [28, 29]. In early infection, exponential rise in HIV-1 viral level parallels a rapid decline in CD4^+^ T-cells. The loss of a protective cellular barrier compromises the intestinal epithelial barrier resulting in increased gut permeability and migration of gut microbiota [2, 12, 30, 31].

During the progression of HIV-1 infection, translocation of gut microbes parallels the elevation of microbial by-products such as lipopolysaccharide (LPS), which activate T-cells and induce pro-inflammatory immune responses. Excessive T-cell turnover causes accumulation and activation of CD8^+^ cytolytic T lymphocytes (CTL)-cells. HIV-1-specific CD8^+^ CTLs secrete cytokines, such as IFN-γ, TNF-α, and IL-2, to promote infected cell death. HIV-1 specific CD4^+^ T helper (Th) cells also produce cytokines to maintain effective CTL activity [32]. These immune responses directly damage the lymphoid tissue and cause immune dysfunction [33, 34]. Together, these processes lead to immune cell senescence and increased soluble immune mediators, including CRP, IL-6, and TNF-α, which do not normalize in PWH despite being treated with ART [2], [3], [4, 35], [36], [37]. Persistent viremia in HIV-1 targeted sites is thought to be responsible for chronic systemic inflammation widely associated with HIV-1.

While no single mechanism can fully capture the molecular and cellular processes underpinning HIV-associated inflammation, there is growing literature demonstrating the role of purinergic receptor signaling and the NLRP3 inflammasome pathway in HIV-1 infection and its associated inflammatory response [2, 3, 35, 36, 38]. Purinergic receptors are ubiquitous in mammalian cells, and the NLRP3 inflammasome is a vital part of the innate immune system [39–41]. They mediate immune function and cell signaling by detecting metabolic stress signals. The P2X purinergic receptors are ATP-gated non-selective cation channels found on various tissues, including immune cells [42–44]. When activated, the P2X receptors act in concert with Toll-like receptors (TLR) to activate the NLRP3 inflammasome. The NLRP3 inflammasome, in turn, activates caspase-1, leading to the maturation and secretion of pro-inflammatory cytokines IL-1β and IL-18 and pyroptosis, a programmed cell death [40, 45, 46]. Several P2X-selective antagonists block HIV-1 infection by inhibiting virus-cell fusion and HIV-induced inflammatory cytokine production, thereby supporting their role in HIV-1-associated infection and inflammation [2, 3, 35, 36, 38].

### The therapeutic impact of cannabinoids in PWH

Recreational and medicinal cannabis use is common among PWH. In the 2005–2015 National Survey on Drug Use and Health (NSDUH), more than one-third (34.9%) of PWH reported cannabis use, about three times greater than in the general population in the US (13.3%) [47–50]. This higher rate of cannabis use in PWH has been attributed to managing HIV-1-associated symptoms, including chronic pain, nausea, and appetite loss, and alleviating emotional unsteadiness, such as anxiety and depression [47, 51], [52], [53], [54], [55]. Medical cannabis is now legal in nearly half of the US, and HIV-1 infection and chronic pain are approved conditions for medical marijuana use [51, 55, 56].

The immunomodulatory properties of cannabis are well-established and have been extensively studied in numerous diseases associated with chronic inflammation [57, 58]. The Δ^9^-tetrahydrocannabinol (THC), the psychoactive component of *Cannabis sativa*, is the most extensively studied cannabinoid. THC exerts its effect through cannabinoid type 1 and 2 receptors (CB1R and CB2R, respectively). Recent literature has shown that THC inhibits T-cell responses *in vitro* by suppressing IL-2 production and stimulating cAMP, which blocks T-cell receptor signaling and proliferation [57–59]. THC also impacts the overall cytokine production profile by down-regulating pro-inflammatory Th1 cytokines, including IL-1β, IL-2, IL-12, and IFN-γ, and up-regulating anti-inflammatory Th2 cytokines, including IL-10 and TGF-g [60, 61].

Several studies have demonstrated the protective roles of cannabinoids in HIV-1 pathogenesis. For instance, synthetic nonselective cannabinoid agonists WIN55,212-2 and CP55,940 have been shown to reduce HIV-1 expression in CD4^+^ T-cells and microglia *in vitro* in a concentration and time-dependent manner [62, 63]. WIN55,212-2 and ACEA, a selective CB1R agonist, have also been shown to inhibit HIV-1 Tat-induced inducible NO synthase (iNOS) protein expression and nitrite production in rat C6 glioma cells [64]. This CB1R-specific inhibition of the NO pathway in microglia reduces cytotoxicity and inflammation associated with HIV-related neurodegeneration [64]. Activation of CB2R has also decreased cell-free and cell-to-cell transmission of CXCR4-tropic HIV-1 virus in primary CD4^+^ T-cells [65].

Cannabinoids have also been investigated for their neuroprotective roles. For example, blockade of the endocannabinoid hydrolytic enzyme, fatty acid amide hydrolase (FAAH), down-regulates Tat-mediated intracellular calcium, neuronal death, and dendritic degeneration in the murine primary neuronal culture model [66]. Similarly, monoacylglycerol lipase (MAGL) inhibition via CB2R prevents gp120-induced synapse loss and decreases gp120-induced IL-1β production and NMDA receptor-mediated calcium influx [67]. Cannabinoids have been shown to restore the integrity of gp120-mediated injury in the blood-brain barrier in mice and cell culture models [68].

Studies on non-human primate models have also provided evidence for cannabinoids exerting immunoprotective roles in simian immunodeficiency virus (SIV) infection. SIV-infected rhesus macaques chronically treated with THC were shown to have reduced plasma and cerebrospinal fluid SIV viral load with decreased early mortality and attenuated progression of SIV disease [69, 70]. Chronic THC treatment of SIV-infected rhesus macaques has also been shown to inhibit classical pro-inflammatory miRNA and gene expression in the GI tract and prevent peripheral lymph node fibrosis [71–73]. THC suppresses intestinal T-cell proliferation and programmed death-1 (PD-1), increases intestinal CD163 anti-inflammatory macrophages, and reduces CD8^+^ T-cell expansion in peripheral blood, all of which contribute to suppressing SIV-associated intestinal inflammation [71].

Studies with human subjects have been more challenging in establishing a definitive causal role for cannabinoids in HIV-1 infection and associated inflammation. Some reasons for this include the following: many studies are observational in nature, there is often a small sample size of studies, individuals are followed for short durations, and a key confounding variable is the high rate of polysubstance use among cannabis users. One observational study showed that PWH who are heavy cannabis users exhibit lower frequencies of activated T lymphocytes and inflammatory antigen-presenting cell (APC) subsets and reduced production of pro-inflammatory cytokines compared with PWH who are non-users [58, 74].

### Endocannabinoids and inflammation

**Figure 1:**
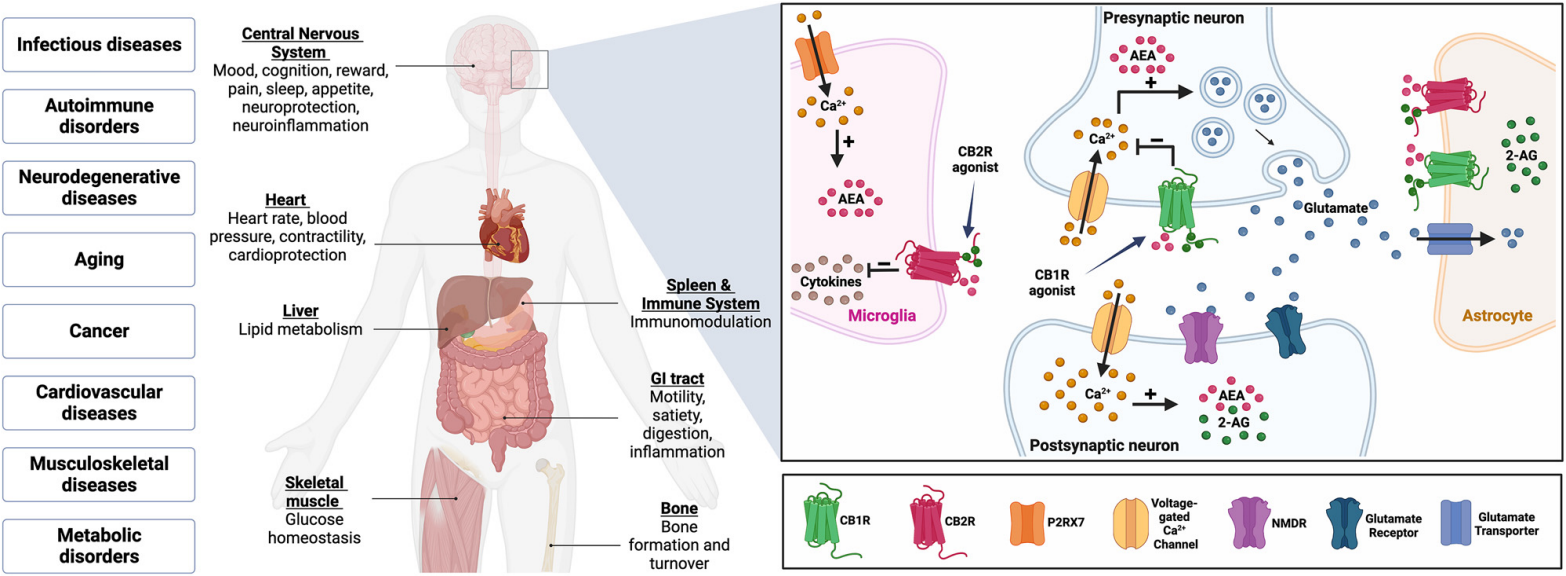
The role of cannabinoid receptor signaling in physiological functions and pathological states (left). Magnified image to the right is a schematic representation of the endocannabinoid system in the brain.  CB1R are expressed by presynaptic neurons and are activated by endocannabinoids, AEA and 2-AG. Calcium (Ca^2+^) influx into the presynaptic neuron leads to release of excitatory glutamate that either binds to postsynaptic receptors, NMDR or glutamate receptor, or gets taken up by glutamate transporters on astrocytes. CB1R agonists block glutamate release and excitotoxicity, which is linked to neuroinflammation. CB2R is predominantly expressed by microglia. CB2R agonists reduces neuroinflammation by blocking the production of proinflammatory cytokines.  Activation of selective purinergic receptor P2RX7 leads an increase in 2-AG production. Created with BioRender.com.

The endogenous cannabinoid was first identified in the late 1980s [75]. These compounds are hydrophobic lipid messengers that are derivatives of integral components of the phospholipid bilayers of cellular membranes [76]. The best-characterized endocannabinoids are anandamide (AEA) and 2-arachidonoylglycerol (2-AG) [76–78]. These molecules are produced on demand in response to external stimuli. Cannabinoids mediate their effect by binding to two major G protein-coupled receptors, CB1R and CB2R [58, 76], [77], [78]. CB1R and CB2R are expressed in most tissues, but CB1R is predominately found in central nervous system (CNS), whereas CB2R is abundant in immune cells (including macrophages, microglia, monocytes, and T-cells), thymus, tonsils, among others. Indeed, CB2R is highly expressed by microglia, the resident macrophages of the CNS, and have gained attention as a potential therapeutic target [78]. Figure 1 demonstrates an overview of all the systemic disease states and physiology that are impacted by cannabinoid signaling, focusing on the CB1R and CB2R receptor signaling in the brain.

Activated cannabinoid receptors lead to specific signal transduction that turns on genes regulating cell migration and the production of cytokines and chemokines [77]. CB1R is typically coupled to particular ion channels associated with adenylate cyclase (AC) and thus affects the level of intracellular cAMP [79]. Their downstream effects include hypokinesia, catalepsy, and analgesia. CB2R has been shown to modulate cytokine release and immune cell migration. For example, binding of 2-AG to CB2R has demonstrated immunomodulatory properties [80]. 2-AG suppresses the lymphoproliferation of splenocytes when exposed to bacterial LPS. It has also been shown to induce chemotaxis, a directed movement of cells toward a gradient of a chemical stimulus, in human eosinophilic leukemia EoL-1 cells and human peripheral blood eosinophils in a CB2R-dependent manner [77].

Endocannabinoids elicit localized effects that are relatively short-lived and stimulatory in nature [76]. In the brain, acute injury or an inflammatory stimulus cause resident CNS macrophages to proliferate and produce pro-inflammatory cytokines, such as IL-1b, IL-6, IL18, and TNF-α. Pro-inflammatory factors contribute to the breakdown of the blood-brain barrier (BBB) and migration of immune cells into the CNS from the periphery [76]. Cannabinoids act to inhibit this inflammatory response. THC and CP55940 have been reported to inhibit the recruitment and migration of immune cells [81–86]. CB2R expression has been shown to be upregulated by microglia and other immune cells during neuroinflammatory states. Hence CB2-selective agonists may have a promising role in counteracting neuroinflammatory diseases [76].

### Endocannabinoids and HIV-1-associated neuroinflammation?

The role of CB2R has also been implicated in HIV-1-associated neuroinflammatory pathogenesis [55, 87]. In the healthy brain, CB2R expression is limited, whereas CB2R expression by microglia and resident CNS perivascular macrophages is high during inflammatory states, such as in HIV-1 or SIV infections [88]. PWH with encephalitic disease demonstrated increased CB2R expression by microglia, perivascular macrophages, and endothelial cells, while people without HIV-1 had nearly undetectable CB2R expression [89]. This increased CB2R expression due to an inflammatory stimuli is likely a negative feedback response to counteract the ongoing inflammation. Hence CB2R agonists have the potential to downregulate inflammation [87]. In surgically resected human temporal lobe tissue, JWH-015 (1-propyl-2-methyl-3-(1-naphthoyl)indole), a selective CB2R agonist, has been shown to decrease microglial inflammation and cytotoxicity, increase cell viability, and lower IL-1β and TNF-α secretion [90, 91].

HIV encephalitis (HIVE) is associated with progressive memory loss, behavior changes, intellectual deterioration, and motor deficits. It is characterized by brain damage due to a surge of pro-inflammatory cytokines, and glutamate neurotoxicity. HIV-1 gene products such as the transactivator tat and the envelope glycoprotein gp120 released from infected monocytes and microglia also add to the insult by increasing glutamate release and subsequently causing neuronal loss [48]. Studies on macaques with SIV-induced encephalitis have suggested that the endocannabinoid system plays a role in the pathophysiology of HIVE [92]. It has been proposed that CB2R activation by immune cells reduces their antiviral response and favors entry into the CNS. It has also been reported that CB2R activation cause inhibition of transendothelial migration of Jurkat T cells and primary human T-lymphocytes by interfering with the CXCL12/CXCR4 chemokine receptor system [93]. This suggests that activation of CB2R can alter the activation of other G protein-coupled receptors, such as CXCR4, which functions as a co-receptor for T-lymphotropic HIV-1. A similar observation was made between CB2R and CCR5, which acts as co-receptor for monotropic HIV-1, indicating that G protein-coupled CB2R can crosstalk with other G protein-coupled receptors, mainly chemokine receptors, and consequently affect heterologous signal transduction pathways [76].

**Figure 2:**
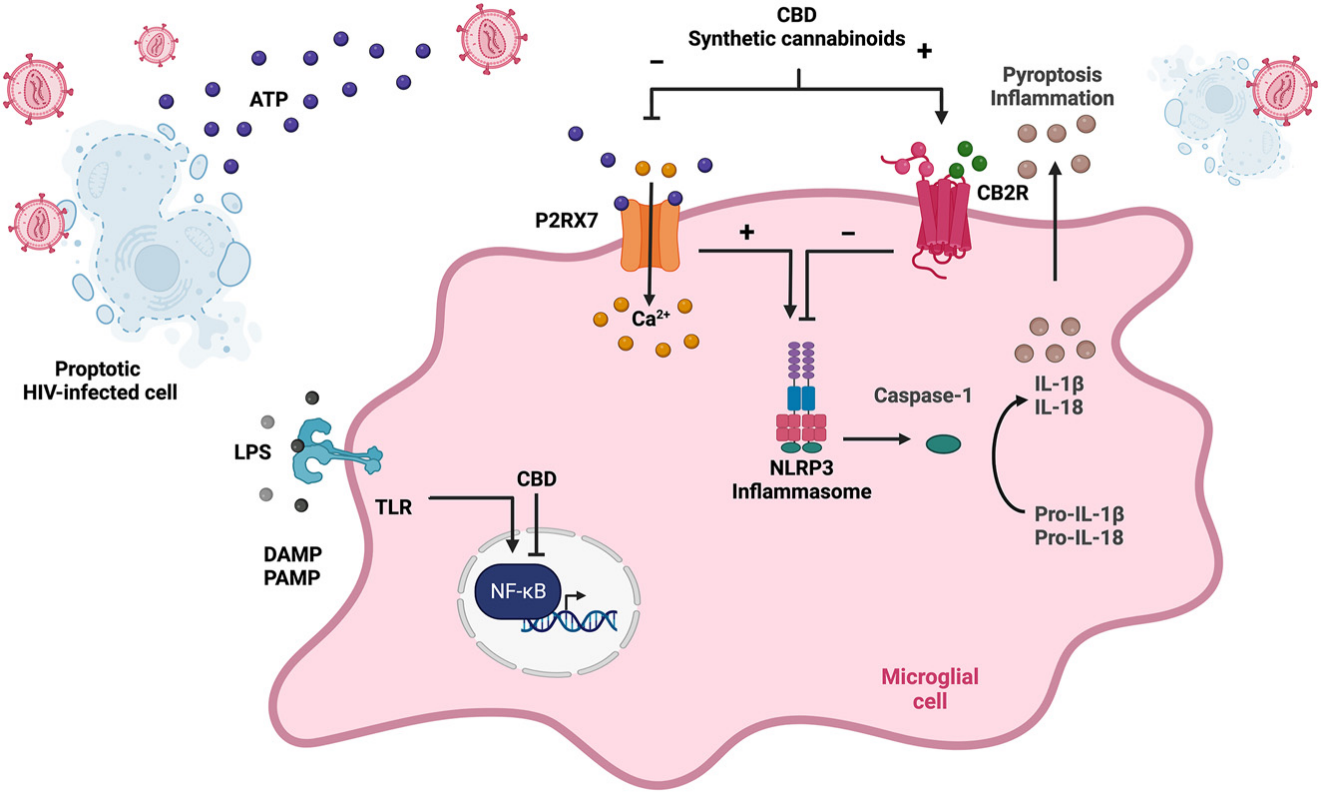
A microglial cell is activated during acute HIV-1 infection and CBD and synthetic cannabinoids can modulate downstream signal transduction. A pyroptotic cell is shown nearby with extracellular ATP signaling through the P2RX7 receptor. Subsequent Ca2+ influx, coupled to toll-like receptor (TLR) signaling by lipopolysaccharide (LPS), activate the NLRP3 inflammasome. Activated NLRP3 inflammasome releases caspase-1 that cleaves precursor pro-IL-1b and pro-IL-18 to produce mature IL-1b and IL-18. Secretion of IL-1b and IL-18 lead to pyroptosis and inflammation. CBD and synthetic cannabinoids activate CB2R expressed by microglia and antagonize the NLRP3 inflammasome via P2RX7. Created with BioRender.com.

Inflammasome activity contributes to the chronic, pro-inflammatory state in PWH. The inflammasome is a part of the innate immune system activated by pattern recognition receptors, notably toll-like receptors (TLR) and purinergic receptors that detect extracellular ATP [2, 94], [95], [96], [97], [98], [99], [100]. The inflammasome activates caspase-1, which cleaves prointerleukin-1β (pro-IL1β) into the mature, secretory interleukin-1β (IL-1β). Inflammasome activation also mediates programmed cell death of myeloid and lymphoid cells or pyroptosis. HIV-1 infection can induce inflammasome activation as intermediary products are recruited by the DNA sensor IFI16 to signal through NLRP3 with an additional signal such as toll-like receptor activation [101, 102]. Monocyte-derived macrophages primed with HIV-1 have increased IL-1β production after exposure to the second NLRP3 signal activators. Specifically, HIV-1 virions induce Toll-Like Receptors (TLRs) to induce pro-IL-1β expression [103, 104]. Active HIV-1 infection is required to activate this pathway, as when exposed to ARVs, induction of pro-IL-1β and release of IL-1β were decreased [103]. ASC speck protein is a marker for inflammasome activation, suggesting that in PWH, activation of pyroptotic cell death is responsible for progressive CD4 T-cell death and may contribute to a chronic inflammatory state. Inflammasome activation persists in immune non-responders, defined as patients on ART without CD4 recovery (CD4 < 350) [105]. When stimulated with LPS, upregulation of inflammasome genes (NLRP3, caspase 1) was seen in both immune non-responders and patients with the immune response (CD4>500). Substance use can further enhance inflammasome activation in PWH. Exposure of cocaine to HIV-1 infected macrophages increases activity by priming the NLRP3 inflammasome by potentiating reactive oxygen species (ROS) production [106, 107]. HIV-1 activation of the inflammasome has a proposed role in neuroinflammation and nephrotoxicity. HIV-1 both primes and activates the microglial NLRP3 inflammasome leading to increased IL-1β and IL-18, contributing to neuronal apoptosis and neuroimmune activation [108–110]. Figure 2 demonstrates that CB2R receptor signaling can impact inflammasome activation in the setting of HIV-1 infection.

### The impact of cannabis use in PWH

PWH usage of cannabis-derived products is 2–3 times higher than the general population, even in people on ART [58]. Namely, the higher use of cannabis-derived products for self-treatment of chronic inflammation-related symptoms is well-established among PWH. In one randomized clinical trial, smoking cannabis at a high but tolerable dose (1–8% THC) yielded a statistically significant decrease in self-reported neuropathic pain intensity in HIV-1-associated diabetic painful distal neuropathy [111]. Meaningful pain relief (previously established as >30% decline in pain) was achieved in the described population (46%). Qualitatively, patients also reported reduced levels of mood disturbance, decreased self-reported physical disability, and improved quality of life with increasing doses of THC. Previous literature has corroborated this anti-inflammatory property of cannabis. For example, the Grant group demonstrated significant differences in the expression of multiple pro-inflammatory biomarkers (IL-6, MCP-1/CCL2, IP-10/CXCL10, sCD14, sTNFR-II, TNF-α) in PWH compared to the general population without HIV-1 [112]. Here, IL-6 has been shown to play an essential role in HIV-1-related inflammation. IL-6 levels have been associated with increased HIV-1 RNA levels and decreasing CD4^+^ T-cell count [113]. PWH who use cannabis daily also demonstrated decreased CCL2 and CXCL10 levels compared to the control population. Related to this, CCL2 levels in the CSF were negatively correlated with learning capability. A non-significant correlation was noted between overall neurocognitive performance metrics (e.g., verbal fluency, memory retention, and motor skills) and cannabis use. PWH who are daily cannabis users seemed to score higher than PWH who did not use cannabis. Another study provided evidence that cannabis usage in PWH was associated with a reduced neurocognitive impairment [114], although this was not the case in the non-PWH population.

PWH has long used cannabis for an evolving list of indications: traditionally for nausea, wasting, and anorexia [54, 115], and more recently for mood symptoms and recreational purposes [116]. There is abundant literature on the numerous immunomodulatory roles of cannabis in HIV-1 [58, 74, 117–120]. Activation of the CB2R receptor has been associated with a reduction in several aspects of HIV-1 infection and associated inflammation, including reduced CXCR4-tropic HIV-1 productive infection, reduced macrophage migration, delayed progression of the simian immunodeficiency virus (SIV)-infected macaques, and reduced inflammatory biomarkers in PWH [58, 65, 85, 121]. Recent studies indicate that cannabis use reduces the activation of CD4^+^ and CD8^+^ T-cells, shifts the monocyte population in peripheral blood, and is associated with faster decay of HIV-1 DNA [74, 118]. The findings that cannabinoid treatment and CB2R activation are associated with reduced HIV-1 infection and inflammation and with reduced NLRP3 inflammasome signaling suggest a link between HIV-1 disease, CB2R receptor signaling, and NLRP3 inflammasome activation. Several studies demonstrate that activation of CB2R is associated with NLRP3 inflammasome inhibition, thus reducing inflammation in myocardial infarction, colitis, and surgery-induced cognitive impairment [122–125].

## Discussion

Chronic inflammation in PWH plays an essential role in overall health outcomes and is associated with neurological, cardiovascular, and many other disorders, as well as cancer. Among the significant causes is chronic inflammation attributed to low-level viremia in stable reservoirs, which cannot be easily accessed by ART, such as the CNS. Elevated levels of T-cell activation and other markers of inflammation persist in these patients despite treatment with ART. A literature review shows that HIV-1 plays a role in priming and activating the NLRP3 inflammasome pathway. As important mediators of inflammatory signaling and cell death, the NLRP3 inflammasome and purinergic receptors have been implicated in chronic inflammation in PWH, and P2X-selective antagonists have been shown to reduce inflammation associated with HIV-1 infection.

Overall, endogenous cannabinoid receptors are an important target in HIV-1-related inflammation. Agonists for cannabinoid receptors show protective effects against virally induced inflammatory responses in multiple animal studies, particularly in the case of CB2R that is primarily expressed in immune cells. CB2R activation is associated with a reduction in HIV-1 replication in CD4^+^ T-cells, as well as decreases in T-cell proliferation and a shift in both myeloid and lymphoid immune populations towards a less inflammatory phenotype. Because of the abundance of CB2R in microglial cells, the main drivers of inflammatory signaling and subsequent BBB breakdown in the CNS, it is particularly implicated as a possible drug target for HIV-1-associated neurocognitive disorders (HAND).

It is evident from the literature that cannabinoids show protective effects against inflammation associated with HIV-1. In both human and animal studies, THC/cannabis treatment has been shown to reduce inflammatory markers, including NLRP3-associated cytokine signaling and T-cell activation and proliferation. Studies also implicate a neuroprotective effect against NO-mediated cytotoxicity and BBB breakdown in rodents. Taken together, these findings suggest a role for cannabinoid receptor activation in reducing chronic inflammation and associated pathologies in PWH. However, further investigation is needed to fully explore the mechanisms underlying the link between HIV-1, NLRP3 inflammasome activation, and cannabinoid receptor signaling.
